# HOME HEALTHCARE WORKFORCE AND NEIGHBORHOOD CHARACTERISTICS

**DOI:** 10.1093/geroni/igac059.1138

**Published:** 2022-12-20

**Authors:** Chenjuan Ma

**Affiliations:** New York University, New York, New York, United States

## Abstract

Despite the rapid increase in the needs for home- and community-based services (HCBS), including home health care which is the most commonly used HCBS, workforce shortage has become a critical challenge to home health agencies in providing quality care to meet the needs of millions of homebound Americans. This study aimed to examine the availability of home health care workforce and its variations by neighborhood characteristics. We linked several national datasets from 2019 and included information from 11,005 HHC agencies in 1,849 counties. The unit for analysis is county. We found that on average county had fulltime equivalent (FTE) 83 (SD=351) home health care nurses, 120 (SD=411) FTE skilled home health providers (e.g., nurses, physical/occupational therapists) and 37 (SD=411) FTE aides. For every 1,000 persons, on average counties had 0.7 (SD=4.6) FTE nurses, 0.9 (SD=4.7) FTE skilled providers, and 0.2 (SD=0.8)) FTE aides. For every 1,000 older adults (>=65), on average counties had 3.6 (SD=23.9)) FTE nurses, 4.8 (SD=24.6) FTE skilled providers and 1.2 (SD=4.4) FTE aides. We also found that counties with moderate (2nd tertile) proportion of Black and Hispanic Americans; counties with highest (3rd title) proportion of Black and Hispanic Americans had the lowest number of FTE home health care aides per every 1,000 persons. Our findings highlight the staff shortage facing home health care and suggest the existence of disparities in availability of home health care workforce.

